# The Effects of Drugging in Life and in Literature

**Published:** 1914-04-11

**Authors:** 


### SOME DANGERS OF DRUG HABITS.

### The Effects of Drugging in Life and in Literature.

It is seldom that a week passes without there being recorded in considerable detail cases in which people who have habitually dosed their systems with excessive quantities of hypnotic drugs overstep the limits wherein safety, to life at least, is possible and perish miserably as a result of their indiscretions. There is, of course, the alternative suggestion of *felo de se* in many of these cases, but coroners' juries, with the kindliness which characterises those much-abused bodies, usually prefer to give the unfortunate ones the benefit of the doubt and return a verdict of death by misadventure. Experience goes to prove, however, that in the majority of cases the fatal result is brought about with full intent.

But death is not the worst sequel for the drug-taker in many cases. Indeed, it is for him a rest from his grievous troubles. But there is much to be endured before that consummation is reached: like that unfortunate poet Cowper, he also will but too frequently "lie down in horror and rise up in despair." There is the steady diminution of resistive power, the physical deterioration, the cry of the tissues for more and more of the stimulant, and in many cases the gradual moral degradation which shows itself so markedly in the steady failure of altruism. It is, of course, true that in many cases the taking of drugs is but a symptom of an unstable nervous organisation. There are numbers of people who are able, for instance, to abstain from alcohol for weeks or months without effort, and to whom indeed during such times the taking of alcohol is unpleasant; the nervous crisis, however, recurs, and the craving once more asserts itself and compels them to obtain the necessary stimulation of their tissues at any cost. Then what is termed a vicious circle is instituted; the unbalanced brain insists on fresh supplies of the chosen stimulant, and this in its turn further weakens the defences of the body until grave results may be brought about. The chronic alcoholic lives on the borderland of that appalling condition, *delirium tremens*, and he may be precipitated into an attack of this kind as a result of a more than usually heavy bout of drinking, or by some bodily injury which overturns the extremely unstable equilibrium into which he has reduced his nervous system.

There are other sequels, however, to chronic alcoholism. After long periods of alcoholic excess certain persons develop delusionary ideas; and, whilst the memory for recent events is frequently bad, there is a recollection of, or rather a formulation of, incidents to replace these.

Opium and its derivatives, and of the latter morphia in particular, have gained quite a place of their own in specialist and in general literature. De Quincey drank laudanum as freely almost as many people drink port, though his was by far the more expensive taste. He has depicted vividly some of the results in his case: the calling up of memories of past events and of recollections of what he had read, the whole recurring in a chaotic and for the most part disorderly way; whilst the unusual stimulation of the brain-substance evoked unsuspected stores of knowledge which otherwise would have remained dormant. The visions, or hallucinations, are, however, entirely dependent on the individual's previous experience; and those of a De Quincey are very different from those of an illiterate Chinaman smoking his pipe in an opium den. It is much to be regretted that any sort of glamour should be cast over such a habit as the taking of opium or of morphia: the results are dire, and no transient pleasure can atone for them. There is with these drugs, too, the degradation which has been noted in regard to the persistent taking of alcohol in large quantities; and with this difference, that it is brought about more rapidly. Mr. Robert Hichens has well described the characteristics of the morphinist in that remarkably able, if somewhat depressing, book "Felix"—a book undoubtedly based upon shrewd study. In the early stages little change may be noted, but as time goes on there is usually an imperative craving for increased and more frequent doses; without the drug there are feelings of intense irritability and restlessness, which can only be alleviated by recourse to fresh injections. Then occur, however, in the course of time, sleeplessness, impaired nutrition, and other symptoms which may lead the sufferer to end it all and escape from his troubles and temptations by means of suicide. He may, like the alcoholic, be haunted by imaginary beings who threaten him, and heap abusive epithets upon him; he becomes a liar, and it is practically an axiom among those who have to deal with the confirmed morphinist that he is certain to be untruthful even when there is no apparent benefit to be derived from the lie; and finally, everything is subordiated to his desire for the drug, and he becomes slavishly dependent on it. Thus the statements of the opium-taker or of the morphinist must be accepted with extreme caution, even in regard to the quantity of the drug which is being taken, which they will frequently boastfully exaggerate, when they do not deny the habit altogether. The present writer has, however, seen a lady of twenty-six years of age empty twenty-five grains of morphia into the palm of her hand and straightway swallow the whole quantity. There were apparently no untoward effects. It may be noted, in passing, that the ordinary dose is about one-quarter or one-half of a grain; so that some idea may be obtained of this particular patient's previous habits when so marked a toleration of the drug had been arrived at.

Cocaine, another drug which is taken for its stimulating effect, though not so frequently as is morphia, is even more potent in its effects: the general physical and mental deterioration is more rapid. Hypnotic drugs are naturally, in such an age of extreme mental concentration and of nervous tension, frequently in demand; and in what are termed the synthetic products, such as sulphonal and veronal, there has lately been intense popular interest. Apparently a habit may be formed in regard to any drug, no matter how nauseous and unpalatable it may be; a certain effect is obtained, and this seems to be sufficient to atone for other effects which are unpleasant and noxious. With such a drug as veronal, however, there are not any markedly unpleasant symptoms in the majority of cases; and it is certainly efficient in producing an unconsciousness which seems to resemble fairly closely natural sleep. Yet with all such remedies which, administered in small quantities, are able to produce a marked and speedy effect, there must be danger when they are in the hands of careless or of neurotic persons. Untoward results occasionally follow even after comparatively small doses; it would, therefore, seem to be advisable that the administration of such potent substances should be relegated to those possessing the requisite knowledge, and the law should be so amended that the murderer, the would-be-suicide, and the retail vendor are still further hampered in procuring or in selling these noxious materials.

